# Patterns, trends and sex differences in HIV/AIDS reported mortality in Latin American countries: 1996-2007

**DOI:** 10.1186/1471-2458-11-605

**Published:** 2011-07-29

**Authors:** Monica Alonso Gonzalez, Luise Martin, Sergio Munoz, Jerry O Jacobson

**Affiliations:** 1 Pan American Health Organization. HIV/STI project Washington DC, USA; 2 DAAD (German Academic Exchange Service), Carlo Schmid Program, Bonn, Germany; 3 Facultad de Medicina, Universidad de La Frontera de Chile, Chile; 4 Pan American Health Organization. HIV/STI project. Bogota, Colombia

## Abstract

**Background:**

International cohort studies have shown that antiretroviral treatment (ART) has improved survival of HIV-infected individuals. National population based studies of HIV mortality exist in industrialized settings but few have been presented from developing countries. Our objective was to investigate on a population basis, the regional situation regarding HIV mortality and trends in Latin America (LA) in the context of adoption of public ART policies and gender differences.

**Methods:**

Cause of death data from vital statistics registries from 1996 to 2007 with "good" or "average" quality of mortality data were examined. Standardized mortality rates and Poisson regression models by country were developed and differences among countries assessed to identify patterns of HIV mortality over time occurring in Latin America.

**Results:**

Standardized HIV mortality following the adoption of public ART policies was highest in Panama and El Salvador and lowest in Chile. During the study period, three overall patterns were identified in HIV mortality trends- following the adoption of the free ART public policies; a remarkable decrement, a remarkable increment and a slight increment. HIV mortality was consistently higher in males compared to females. Mean age of death attributable to HIV increased in the majority of countries over the study period.

**Conclusions:**

Vital statistics registries provide valuable information on HIV mortality in LA. While the introduction of national policies for free ART provision has coincided with declines in population-level HIV mortality and increasing age of death in some countries, in others HIV mortality has increased. Barriers to effective ART implementation and uptake in the context of free ART public provision policies should be further investigated.

## Background

The worldwide scale-up of antiretroviral therapy (ART) has substantially improved survival and quality of life of HIV-infected individuals. There is clear evidence that mortality associated with AIDS as well as incidence of AIDS-defining clinical conditions have fallen drastically in industrialized countries that provide good access to ART, such as the United States and Western European countries [1-9]. Improvement in survival among AIDS patients in developing countries has also been documented [10-12].

Despite increases in ART coverage to more than 4 million people in low- and middle-income countries by 2008 [13], HIV/AIDS has continued to be the fourth most frequent cause of death in low income countries [14]. Greater evidence is needed to better understand the impact of the HIV epidemic and AIDS mortality following the introduction of ART.

Models for the HIV epidemic for Latin America (LA) have shown a 21% increment in HIV annual mortality between 2001 and 2004 and a drop to 16% in the year 2008 [15]. The decline between 2004 and 2008 has been associated with increased availability and access to ART throughout the Latin American region [15]. Yet, differences both among and within countries exist and treatment coverage continues to be suboptimal in many areas [16-18].

Studies including cohorts from Latin American countries have shown improvements in survival among patients on ART [16,19]. In addition to HIV cohorts, the study of vital registration data and reported mortality due to HIV/AIDS can provide insight regarding the impact of HIV programs and policies at the population level. Several countries in LA have conducted trend analyses of reported HIV mortality [20-31]. Here, we provide a regional analysis of HIV/AIDS mortality between 1996 and 2007, in the context of adoption of public policies marking commencement of national provision of ART between 1997 and 2004 in Latin American countries with mortality records classified from average to good quality. The objective was to analyze patterns and trends in population-level mortality due to HIV/AIDS among Latin American countries and compare age-adjusted trends in mortality by sex.

## Methods

This study draws on national vital registration mortality data from Latin American countries for the available years at the time of analysis using ICD10-coded data. Only countries with "good" or "average" quality mortality registration data, according to the Pan American Health Organization (PAHO) [32,33], were examined. The classification for quality of mortality data relies on a composite index based on the proportion of estimated mortality under-registration and on the proportion of deaths assigned to ill-defined and unknown causes. The index was calculated for each country for the years 2003 to 2005, using the formula:

Countries were classified according to the value of the index as having *good quality *(index ≤ 10%; Argentina, Chile, Cost Rica, Guatemala, Mexico, Uruguay, and Venezuela), *average quality *(11 to 20%; Brazil, Colombia, Ecuador, El Salvador, and Panama), *poor *or *very poor quality *(21 to 40%, and > 41%; Nicaragua, Peru, Bolivia, Paraguay) data. No data was available for Honduras and Guatemala was excluded because mortality data was only available for the years 2005 and 2006.

### Data sources and analysis

HIV/AIDS mortality data were obtained from the PAHO database of reported mortality data [34] by cause of death, age and sex. This information is produced by national vital statistics departments, registries and ministries of health and is reported annually by ministries of health in LA to PAHO.

Crude mortality rates were calculated using the 2006 population estimates of the Division of Population of the United Nations as denominators. The numerators were the number of registered deaths for which the underlying cause of death was "Acquired Immunodeficiency Syndrome (AIDS)", corresponding to ICD-10 codes: B20 to B24. No correction for mortality under-registration or redistribution of causes was undertaken.

Poisson regression models were fitted by country to obtain adjusted rate ratios by year, age group and sex, using as baseline the year of commencement of public provision of antiretroviral therapy, as well as to test differences in time trends. When statistically significant differences in trends by sex were detected, using likelihood ratio tests, separate models for men and women were fit and tendencies by sex were presented separately. Processing and analysis of the data was carried out using STATA SE 11.0 for Windows (Stata Corporation, College Station TX, USA).

Age group categories used for standardization of death rates and for regression models were 0 to 4, 5 to 14, 15 to 24, 25 to 44, 45 to 64, and 65 years and over. Mean age at time of death was determined using mean mid-age-group data at time of death. These data were available in aggregated one-year age groups from 1 to 4 years in age and in five-year age groups thereafter. Comparisons of means were conducted using analysis of variance (ANOVA) when comparing by sex and year. Cases of unknown sex or age were included for the analysis of total mortality rates.

In 1996, Brazil became the first developing country to commit to and implement policies to provide free and universal access to highly active antiretroviral therapy (ART). Currently all countries in Latin America have established public policies that provide ART free of charge within designated publicly financed health service facilities. For purposes of the study, we defined the year when this occurred as the year when the national government first published a document delineating a national policy of provision of free of charge ART to all patients in need of treatment [35-39]. All countries included in the study had national ART guidelines that defined first-line regimens. The level of implementation of national guidelines is thought to be uneven within and among countries due to treatment costs, barriers to HIV testing and limitations in health services capacity in each country [40], yet is not known with precision. National estimates of ART coverage between 2004 and 2009 appear in Table 1.

**Table 1:** Population, number and HIV prevalence, ART coverage, and establishment of national policy for public provision of ART (year), % of male deaths, HIV mortality rate ratios in men compared to women 1996-2007

Country	Country population 2009 (in thousands)	Number of persons living with HIV: Total/females 2001/2009	15-49 years old estimated HIV prevalence (%) 2001/2009	Estimated ART coverage (%) 2004**	Year of National ART Policy	% male deaths	Male-female HIV mortality rate ratio *** 95% confidence interval
Argentina	40,666	110000/36000	0.4/0.5	88	1997^1^	74.0%	2.8 (2.7-2.9)

Brazil	195424	NA	[0.3-0.5]/[0.3-0.6]¥	[70-95] ¥	1996^2^	70.1%	2.3 (2.3-2.4)

Chile	17134	40000/12000	0.3/0.4	72	2001^3^	87.45%	6.9 (6.3-7.6)

Colombia	46299	160000/50000	0.8/0.5	20	1999^4^	81.1%	4.3 (4.1-4.4)

Costa Rica	4641	9800/2800	0.2/0.3	95	1997^5^	84.8%	5.6 (4.8-6.4)

Ecuador	13772	37000/11000	0.5/0.4	10	2003^6^	82.6%	4.7 (4.4-5.1)

El Salvador	6193	34000/11000	0.8/0.8	23	2004^7^	72.3%	2.6 (2.4-2.8)

Mexico	110647	220000/59000	0.3/0.3	46	2003^8^	83.8%	5.2 (5.0-5.3)

Panama	3507	20000/6300	1.4/0.9	25	2001^9^	75.0%	3.0 (2.8-3.2)

Uruguay	3373	9900/3100	0.4/0.5	95	1997^10^	76.4%	3.2 (2.8-3.8)

Venezuela	29044	NA	NA	NA	1999^11^	81.7%	4.5 (4.3-4.7)

Source column #1: UN Population projections. Source columns #3, 4 and 5: UNAIDS. Report on the global AIDS epidemic 2010, Geneva, 2010

* Data unavailable for: Panama for 2005; Uruguay for 2002.

ART: antiretroviral therapy

IRR = Incidence rate ratio

** ART coverage using as numerator persons reported on treatment at end of reporting year and denominator the estimated treatment need criteria CD4 < 200.

1. National UNGASS report-Argentina, 2008

2. National UNGASS report -Brasil, 2008

3. National UNGASS report -Chile, 2008

4. OPS/ORAS/ONUSIDA Análisis de la situación de VIH en la subregión andina, 2003-2008, March 2007.

5. National UNGASS report -Costa Rica, 2008

6. National UNGASS report -Ecuador, 2008

7. National UNGASS report -El Salvador, 2008

8. National UNGASS report -México, 2008

9. National UNGASS report -Panamá, 2008

10. Ministerio de Salud Publica. Resolution Nª171/1997, that includes universal access to ARV drugs to all AIDS patients. Uruguay 1997

11. PAHO. Legislation and milestones on access to treatment in Latin America and the Caribbean. Pan American Health Organization. August 2003. Last accessed 30/1/2010. Available at http://www.paho.org/Spanish/AD/FCH/AI/acceso-arv-06.doc

*** Ratios adjusted for age groups and over time for the study period, p < 0.000 for all rate ratios.

NA: Not available

¥: point estimate unavailable for Brazil, modelled uncertainty interval provided

To compare HIV mortality trends among countries, age-standardized mortality rates were calculated for each year using the direct standardization method. The sum of the population estimates for the year 2000 from the Division of Population of the United Nations for the countries included in the study was used as the reference population. The ratio of standardized mortality rates for men relative to women adjusted for age was estimated for each country over the time period to summarize differences by sex.

## Results

HIV/AIDS mortality rates were analyzed for the years 1996 to 2007. Most countries had available data for the years 1997 to 2006. The mean mortality follow up was 9.9 years, with a range of 7 years (for Uruguay) to 12 years (for Brazil) (Table 1). The mean crude HIV mortality rate over the study period was 5.3 per 100,000 population (95% CI 4.7-5.8) with a range of 3.0 to 13.1 per 100,000 across countries.^a^

### Mortality patterns and trends

**Figure 1:**
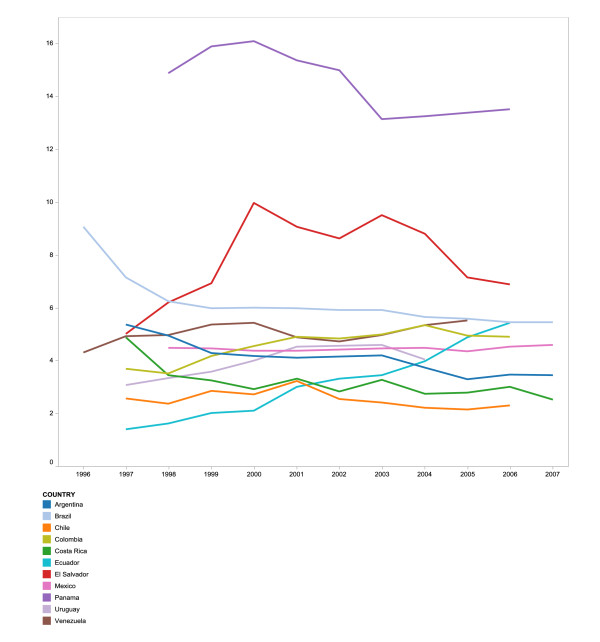
**Sex and age-adjusted HIV mortality rate per 100,000 population**.

Age and sex-standardized mortality rates in the period prior to the adoption of public ART policies in the different countries ranged from 2.2 to 15.6 deaths per 100,000 (mean 5.7, CI 4.1-7.2). In comparison, after the ART public policy adoption the rates were 2.5 to 14.0 (mean 5.1, CI 4.5-5.7; p = 0.4). During the study periods of 1999 to 2004 and 2006, Panama, followed by El Salvador, presented the highest HIV mortality rates and Chile the lowest. Standardization did not alter the HIV mortality rates substantially. HIV mortality trends appear to follow three patterns. About half of the countries present a remarkable decrement in HIV mortality rates after the initiation of public provision of ART (Argentina, Brazil, Chile, Costa Rica and El Salvador). In Panama, the mortality rate decreased after the official start date of public ART provision, but this reduction did not achieve statistical significance. A second group of countries (Colombia and Ecuador) presented a remarkable increment in the HIV mortality trends. Finally, Mexico, Venezuela and Uruguay had varied trends in rate ratios with an overall increasing mortality pattern after adopting the public ART policies (Figure 1). Increase in male and female mortality rates, relative to the year of establishment of free ART public policies, varied from 12% to 39% among countries with an increasing trend, and declined by 15% to 54% for those with a decreasing trend.

**Figure 2:**
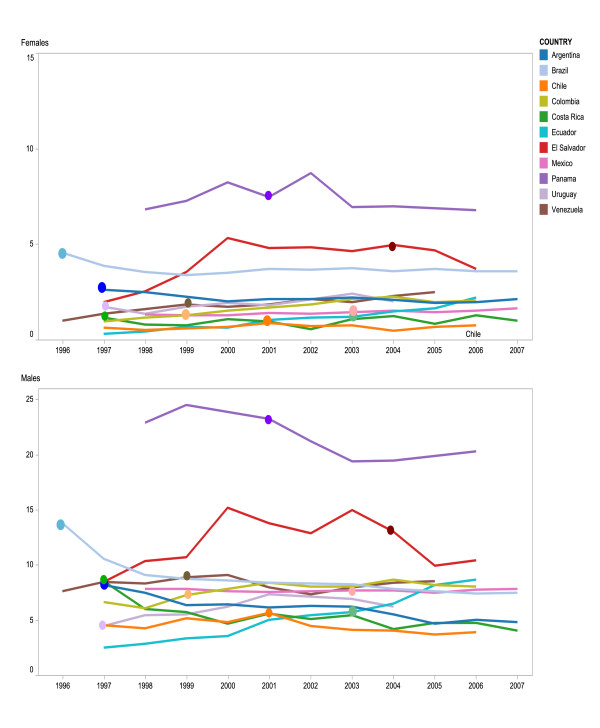
**Age-adjusted HIV mortality rate per 100,000 by sex in Latin American countries, 1996-2007**. Note at bottom of figure: Dot on trends lines mark start year of national provision of antiretroviral treatment.

HIV mortality rates by country were much higher in males when compared to females over the study period, comprising 75% of total HIV/AIDS deaths and with rate ratios from 2.3 to 6.9 relative to females across countries (Table 1) (Figure 2). When evaluating the HIV mortality trends by sex groups significant differences were detected in Argentina, Brazil, Costa Rica, El Salvador, Colombia, Mexico and Venezuela (p < 0.001), not so in Chile, Ecuador, Panama and Uruguay.(Tables 2 and 3).

**Table 2:** Age-Adjusted HIV mortality rate ratios 1996-2007 among countries with significant differences in time trend by sex

	Argentina		Brazil		Colombia		Costa Rica		El Salvador		Mexico		Venezuela	
		F (95% CI)	M (95% CI)	F (95% CI)	M (95% CI)	F (95% CI)	M (95% CI)	F (95% CI)	M (95% CI)	F (95% CI)	M (95% CI)	F (95% CI)	M (95% CI)	F (95% CI)

1996	--	**--**	**1.00**	**1.00**	-- --	-- --	--	--	--	-- --	-- --	--	0.80 (0.73-0.87)	0.52 (0.41-0.65)

1997	**1.00**	**1.00**	0.78 (0.76-0.80)	0.87 (0.83-0.91)	0.87 (0.81-0.94)	0.73 (0.61-0.88)	**1.00**	**1.00**	0.53 (0.45-0.63)	0.34 (0.25-0.46)	-- --	--	0.91 (0.83-0.99)	0.72 (0.58-0.89)

1998	0.93 (0.86-1.0)	0.96 (0.84-1.1)	0.69 (0.67-0.71)	0.81 (0.77-0.85)	0.81 (0.75-0.88)	0.89 (0.75-1.07)	0.73 (0.57-0.93)	0.68 (0.35-1.39)	0.66 (0.57-0.78)	0.44 (0.34-0.59)	0.91 (0.87-0.95)	0.81 (0.73-0.90)	0.91 (0.84-0.99)	0.86 (0.70-1.05)

1999	0.81 (0.75-0.87)	0.87 (0.76-1.0)	0.67 (0.65-0.69)	0.79 (0.75-0.83)	**1.00**	**1.00**	0.71 (0.55-0.90)	0.67 (0.35-1.39)	0.71 (0.61-0.83)	0.63 (0.49-0.80)	0.94 (0.90-0.98)	0.80 (0.72-0.89)	**1.00**	**1.00**

2000	0.83 (0.77-0.90)	0.80 (0.70-0.92)	0.68 (0.66-0.70)	0.83 (0.80-0.87)	1.10 (1.02-1.18)	1.23 (1.05-1.45)	0.60 (0.47-0.78)	0.98 (0.57-1.94)	1.03 (0.90-1.19)	0.98 (0.79-1.22)	0.93 (0.89-0.98)	0.84 (0.76-0.94)	1.05 (0.96-1.14)	0.98 (0.81-1.18)

2001	0.81 (0.75-0.87)	0.87 (0.76-1.0)	0.67 (0.65-0.69)	0.90 (0.86-0.94)	1.20 (1.12-1.29)	1.39 (1.18-1.62)	0.74 (0.58-0.94)	0.86 (0.51-1.78)	0.98 (0.85-1.13)	0.91 (0.73-1.14)	0.94 (0.90-1.0)	0.92 (0.83-1.02)	0.93 (0.85-1.02)	1.06 (0.89-1.28)

2002	0.84 (0.78-0.91)	0.88 (0.77-1.01)	0.68 (0.65-0.69)	0.91 (0.87-0.95)	1.18 (1.10-1.27)	1.54 (1.32-1.79)	0.69 (0.54-0.88)	0.49 (0.26-1.15)	0.93 (0.81-1.07)	0.94 (0.76-1.18)	0.98 (0.93-1.02)	0.93 (0.84-1.03)	0.88 (0.81-0.96)	1.25 (1.04-1.49)

2003	0.86 (0.79-0.93)	0.95 (0.83-1.1)	0.69 (0.67-0.71)	0.95 (0.90-1.0)	1.21 (1.13-1.30)	1.8 (1.55-2.09)	0.75 (0.59-0.95)	1.01 (0.63-2.10)	1.11 (0.97-1.28)	0.93 (0.74-1.16)	**1.00**	**1.00**	0.99 (0.91-1.10)	1.18 (0.98-1.41)

2004	0.78 (0.72-0.85)	0.90 (0.79-1.04)	0.67 (0.65-0.69)	0.93 (0.89-0.98)	1.33 (1.24-1.42)	1.95 (1.68-2.26)	0.60 (0.47-0.78)	1.16 (0.76-2.40)	**1.00**	**1.00**	1.02 (0.97-1.06)	1.07 (0.97-1.18)	1.06 (0.97-1.16)	1.42 (1.19-1.69)

2005	0.69 (0.63-0.75)	0.87 (0.76-1.0)	0.66 (0.64-0.68)	0.98 (0.93-1.02)	1.28 (1.19-1.37)	1.75 (1.50-2.03)	0.69 (0.54-0.88)	0.76 (0.48-1.70)	0.77 (0.67-0.90)	0.98 (0.78-1.21)	1.01 (0.96-1.05)	1.05 (0.95-1.16)	1.11 (1.02-1.21)	1.58 (1.33-1.88)

2006	0.75 (0.69-0.81)	0.89 (0.77-1.01)	0.66 (0.64-.68)	0.97 (0.93-1.02)	1.28 (1.20-1.37)	1.86 (1.60-2.16)	0.71 (0.56-0.91)	1.20 (0.82-2.56)	0.84 (0.72-0.97)	0.79 (0.62-0.99)	1.07 (1.02-1.11)	1.12 (1.02-1.24)	---	--

2007	0.74 (0.68-.80)	0.99 (0.87-1.13)	0.68 (0.66-0.70)	0.99 (0.95-1.04)	-- --	-- --	0.62 (0.48-0.80)	0.98 (0.66-2.17)	-- --		1.10 (1.04-1.14)	1.22 (1.12-1.34)	---	--

HIV mortality rate ratios are calculated relative to the year of commencement of large-scale public provision of ART

* Countries present in this table have statistically significant interactions between sex and year for HIV mortality.

Models adjusted for age groups. Likelihood ratio test for interaction of age with time trend < 0,05 for all countries.

95% CI = 95% confidence interval

**Table 3:** Age-Adjusted HIV mortality rate ratios 1996-2007 among countries with no significant difference in time trend by sex

Year	Chile	Ecuador	Panama	Uruguay
1996	---	---	---	---

1997	0.74 (0.65-0.84)	0.36 (0.30-0.44)	---	**1.00**

1998	0.69 (0.61-0.79)	0.43 (0.36-0.51)	0.89 (0.78-1.02)	1.13 (0.85-1.50)

1999	0.86 (0.76-0.97)	0.55 (0.47-0.64)	0.98 (0.86-1.11)	1.21 (0.92-1.6)

2000	0.83 (0.73-0.94)	0.58 (0.50-0.68)	1.02 (0.90-1.16)	1.39 (1.10-1.80)

2001	**1.00**	0.85 (0.73-0.97)	**1.00**	1.55 (1.20-2.00)

2002	0.80 (0.70-0.90)	0.95 (0.82-1.10)	1.00 (0.88-1.13)	---

2003	0.77 (0.68-0.87)	**1.00**	0.89 (0.78-1.02)	1.63 (1.27-2.11)

2004	0.72 (0.64-0.82)	1.17 (1.03-1.34)	0.92 (0.81-1.05)	1.51 (1.17-1.95)

2005	0.71 (0.62-0.80)	1.46 (1.30-1.70)	---	---

2006	0.76 (0.67-0.86)	1.65 (1.46-1.86)	---	---

HIV mortality rate ratios are calculated relative to the year of commencement of large-scale public provision of ART

Countries present in this table do not have statistically significant interactions among sex and year for HIV mortality rates.

In Argentina, Brazil, Costa Rica and El Salvador, HIV mortality rates among males decreased significantly after the public provision of ART (p < 0.000) (Tables 2 and 3). In contrast, HIV mortality trends among women in those countries either show no decrease, such as in the case of Costa Rica, or show smaller, inconsistent and not statistically significant declines like in the remaining countries. On the contrary, in Colombia, Venezuela and Mexico the HIV mortality rates increased for both men and women (p < 0.000), after ART public provision (Table 2).

In relation with the ART coverage, those countries with high percentage of coverage (more than 70%) had a better HIV mortality outcome. On the contrary, those counties with lower coverage present an increment in their mortality trend (Tables 1, 2 and 3). However Uruguay, with an increasing mortality trend had a high ART coverage (95% in 2004).

### Mortality and age

**Figure 3:**
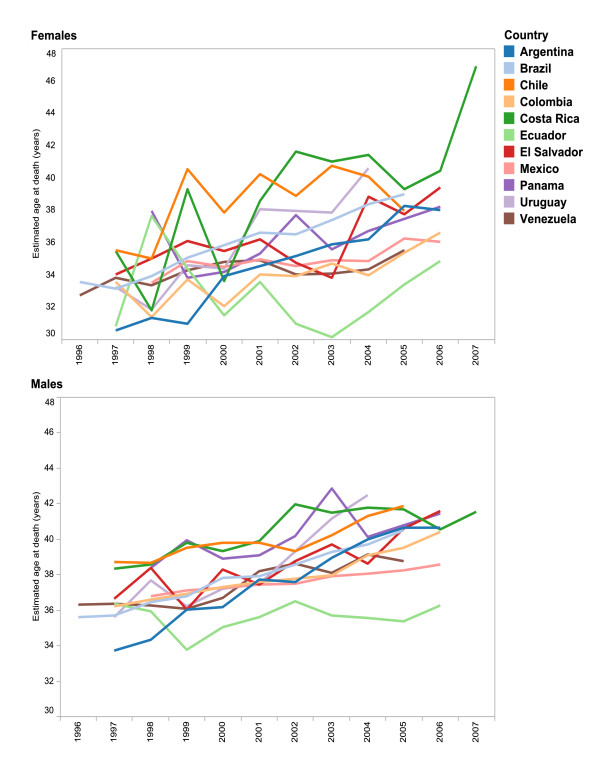
**Mean age at death, due to HIV, by sex, 1996-2007**.

For all countries, the highest mortality rate by age over the period was observed in the 25-44-years age group (p < 0.01)(data not shown). The estimated mean age at death increased over the study period around 2 to 8 years in all countries (p < 0.05), except in Ecuador where an increase in mean age at death occurred only among women. Statistical differences in mean age at death before and after public provision of ART appeared for all countries where mortality data was available (Chile, Colombia, El Salvador, Mexico, Panama, Venezuela) (p < 0.05), with the exception of Ecuador. Mean age at death was consistently lower for women over the period except in Chile and Costa Rica (p < 0.05) (Figure 3).

## Discussion

This is the first article to present population trends in HIV mortality across selected Latin American countries. Results suggest that six out of the eleven countries examined show a decline in HIV mortality after the initiation of national policies providing free public-sector access to ART. The analysis also illustrates how men and women are affected and vulnerable with regards to HIV: adult men had consistently higher mortality due to HIV while women showed less improvement in rates over time and lower mean age at death.

The shift in mean age at HIV death, as measured in our data, ranged from 2 to 8 years. Many factors, including changes during the study period in HIV incidence overall and among specific age groups, an increase in HIV incidence among older adults [41-43], increase in life expectancy among HIV patients, access to and quality of ART, and patients' perceptions of ART availability and effectiveness, are likely to have influenced mortality rates during the period examined by this analysis. Our finding that introduction of national policies for public ART provision coincided with an increase in the mean age of death from 2 to 8 years requires further investigation to better understand the underlying factors. Among many possible explanations, an increase in survival of HIV-infected patients may be related to improved access and quality of ART and in the management and prophylaxis of opportunistic infections, as previously demonstrated in several cohort studies [44-46].

Mortality rates in this study were calculated relative to the census population so that observed changes may capture not only improvements in ART but also changes in epidemic dynamics. For example, decreasing HIV incidence with all other factors stable could, in theory, lead to mortality declines. However, estimated HIV cases and ART treatment caseloads increased, rather than decreased, in all countries included in this analysis during the period [13,47]. Similarly, an increase in the age distribution of incident cases could theoretically explain the increase in age of death observed in this study. Yet, this is unlikely to be the case as younger individuals continue to comprise the majority of reported HIV and AIDS cases [47].

The consistently elevated levels of mortality among men compared to women is most likely explained by the fact that men who have sex with men (MSM) continue to be one of the populations with the highest prevalence throughout Latin America [48]; and may also reflect prevalent gender norms, socioeconomic roles and structures that translate to gender inequities in access to timely diagnosis and treatment in Latin America, including homophobia and *machismo *[49,50].

Differentials in access to HIV services between men and women could contribute to differences in mortality, however the direction of this effect is unclear. Some studies in Latin America have found that women tend to start ART with higher CD4 counts than men, which could be attributed to the scale-up of HIV screening in pregnant women as an entry point to HIV care and treatment. This delay in the HIV detection in the male group could partially explain a higher HIV related mortality. On the other hand, socioeconomic disparities may affect women's ability to access adequate HIV treatments and services more than men both in the US and LA [51-53]. Our findings concurred with these previous publications.

The reduction in HIV mortality rates in the population appears remarkable. This begs the question of how, and how much, HIV mortality could be reduced further? In the United States, the age-adjusted HIV death rate fell by 28% from 1995 to 1996, by 46% from 1996 to 1997, and by 18% from 1997 to 1998. After 1998, the rate leveled off at around 5 per 100,000, which is similar to the level reported in Brazil, Argentina and Costa Rica for the same period [54,55]. Other factors also affect HIV mortality such as the unrelenting occurrence of new infections, lagging ART coverage in some areas, late presentation for HIV care with delayed ART uptake, suboptimal treatment and adherence and lack of treatment options [56]. Specific age, sex and gender identity subgroups may still be experiencing higher HIV mortality even within countries that have substantially lowered their rates. At the regional level this information is not available to conduct more in-depth analysis. National institutions, particularly those where mortality has remained stable or increased, should engage in operational research to identify lost opportunities in the response effort and address inequities in services' access that further reduce HIV mortality.

ART coverage and rate of scale-up, which were not captured in the models, differed among countries and were likely to influence HIV mortality trends. ART coverage may have been too low in some countries to reduce HIV mortality levels. This may be the case in Ecuador where ART scale-up was limited until 2006, when international funds became available [57,58].

Moreover, the rise in HIV mortality seen in some countries may have resulted from a gap in primary and secondary prevention and including late HIV testing and diagnosis, low adherence and issues in treatment quality despite relatively high ART coverage [13,16,46].

An effective universal ART coverage is one goal for controlling the HIV/AIDS epidemic. The introduction of policies that make the access of ART universal and free are key to fulfill this goal, but in reality their coverage varied greatly among the different countries. Furthermore, there are countries with low coverage and this issue could explain the mortality trends differences found in the study.

The principal limitations of the study are associated with the source and quality of the data. Errors in registered mortality could occur during data reporting collection and processing as well as by limitations in medical knowledge, diagnostic errors and deficiencies in the death certification. The validity of the distribution by cause is also affected by under reporting of deaths and cause of death certification, even when conducted by physicians. In many Latin American countries, registration of death is not performed by physicians, particularly in remote and rural areas, which can compromise the accuracy of cause of death classification. Physicians may also prefer certain kinds of diagnoses, introducing biases that may vary from country to country and over time [59]. In addition, legal and societal concerns may lead to the underreporting of causes of a sensitive nature, such as HIV, on the death certificate [59]. Another limitation of the study is the difference in the time of follow up after public ART introduction. Some like Brazil had 11 years of follow up while others, like El Salvador, had only 2 years of follow up post public ART provision.

We also expect some degree of bias due to variation in correct HIV diagnosis and cause of death registration over time. Tendencies to misclassify as well as intentionally replace the registered cause of death may change over time. For example, opportunistic infections and cancers may be more frequently recorded as cause of death in lieu of HIV [7,60-62]. The degree, to which such effects occur, however, is unclear and should be examined further.

## Conclusions

Despite limitations, vital statistics are an important source of information to study the impact of the HIV epidemic at the population level in Latin America and to monitor the joint impact of response efforts. Given potential biases, this information should be analyzed in conjunction with other sources of data, such as HIV case-based reporting, biological and behavioural surveillance, HIV drug resistance surveillance, and coverage of prevention and treatment services in order to adequately monitor the epidemic and its response.

Given the differences by sex found in this study, future assessments of the response to HIV should include a gender perspective and explicitly examine sex and socioeconomic disparities with respect to services, norms and laws that may constitute barriers to service access. Similarly, intervention efforts should seek to involve both men and women as part of an equitable response [63,64]. These results provide useful information to support the monitoring of the epidemic, informing policy and programmatic action, and to motivate efforts to improve the effectiveness of ART implementation and uptake. Further improvement in vital registration data and analysis are needed to improve understanding of the behavior of the HIV epidemic, including sex and gender differences and resulting mortality.

## Competing interests

The authors declare that they have no competing interests.

## Authors' contributions

MAG: Had the original idea, compiled the data, conducted the bibliographical review, wrote the text, prepared the figures and coordinated the overall study. LM: Conducted part of the analysis, prepared the tables, and contributed in writing the text. SM: provided statistical advice and conducted the majority of the statistical analysis. He also contributed to reviewing the text. JOJ: reviewed the text, tables and figures, providing meaningful contributions to the text, analysis and tables and contributed to the bibliographical review. All authors read and approved the final manuscript.

## Pre-publication history

The pre-publication history for this paper can be accessed here:

http://www.biomedcentral.com/1471-2458/11/605/prepub

## Endnotes

^a^Crude rates (not shown) can be requested from authors.
